# Prognostic value of O^6^-methylguanine-DNA methyltransferase hypermethylation and expression in head and neck cancer: A systematic review and meta-analysis

**DOI:** 10.1097/MD.0000000000033472

**Published:** 2022-04-07

**Authors:** Huiwen Yang, Liuqing Zhou, Fan Yang, Jingcai Chen, Yanjun Wang

**Affiliations:** a Department of Otorhinolaryngology, Union Hospital, Tongji Medical College, Huazhong University of Science and Technology, Wuhan, China.

**Keywords:** head and neck neoplasms, meta-analysis, MGMT, O^6^-methylguanine-DNA methyltransferase, prognosis

## Abstract

O^6^-methylguanine-DNA methyltransferase (MGMT) is a DNA repair enzyme that maintains the stability of genetic information. MGMT is a strong prognostic biomarker in patients with glioblastoma. However, the effect of its gene hypermethylation and expression on the survival rate of head and neck cancer (HNC) patients is still disputed. Therefore, we conducted a meta-analysis to evaluate the prognostic value of MGMT hypermethylation and expression in HNC patients.

**Methods:**

This meta-analysis was conducted in accordance with the Preferred Reporting Items for Systematic Reviews and Meta-Analyses 2020 guidelines and was registered at the International Prospective Register of Systematic Reviews (CRD42021274728). Literature related to the survival rate of HNC patients and MGMT was systematically searched in PubMed, Embase, The Cochrane Library and Web of Science electronic databases (published from inception to February 1, 2023). The association was evaluated by the combined hazard ratio (HR) and related 95% confidence interval (CI). Two authors independently screened all records and extracted the data. The certainty of evidence was assessed using the Grading of Recommendations Assessment, Development and Evaluation system. All of the statistical tests used in this meta-analysis were conducted with Stata 12.0 software.

**Results:**

We included 5 studies with 564 HNC patients for the meta-analysis. All of the included patients were primary tumors and underwent surgical resection without prior radiotherapy or chemotherapy therapy. No significant heterogeneity was noted between MGMT and overall survival, MGMT and disease-free survival, and a fixed-effects model was used. HNC patients with MGMT hypermethylation and low expression had a poor prognosis, with pooled HR for overall survival (HR = 1.23, 95% CI: 1.10–1.38, *P* < .001) and disease-free survival (HR = 2.28, 95% CI: 1.45–3.58, *P* < .001). Subgroup analysis stratified by molecular abnormalities, such as hypermethylation or low expression, showed similar results. The insufficient number of trials included in our study encountered high risk of bias and may increase the deviation of the final meta-analysis results.

**Conclusion:**

HNC patients with MGMT hypermethylation and low expression were more likely to exhibit poorer survival. MGMT hypermethylation and low expression can predict survival in patients with HNC.

## 1. Introduction

Head and neck cancer (HNC) are neoplasms of the upper respiratory and digestive tracts, including tumors of the oral cavity, oropharynx, nasopharynx, hypopharynx, larynx, and paranasal sinuses.^[1]^ As the sixth most common cancer globally, more than 800,000 people are diagnosed with HNC each year, and over 400,000 patients die from the disease.^[2]^ In the past 10 years, the diagnosis and treatment of HNC have developed quickly; stage I and II HNC can be cured, but 66% of HNC is diagnosed at an advanced stage, and more than 50% of patients die of metastatic tumors during the 5-year follow-up.^[3]^ But now, by combining immunotherapy, radiotherapy and chemotherapy, it is possible to improve the survival rates of HNC patients. A randomized, open-label study reported that Nivolumab, an anti–programmed death 1 monoclonal antibody, could improve their survival rate and decrease treatment-related adverse events among patients with platinum-refractory, recurrent squamous-cell carcinoma of the head and neck (HNSCC).^[4]^ In addition, another study found that Pembrolizumab improved overall survival (OS) with less adverse events in recurrent or metastatic HNSCC patients who were progressed after platinum based chemotherapy.^[5]^ Immunotherapy has significantly changed the treatment landscape for cancer patients,^[6,7]^ and new immunotherapy targets bring hope to improve the prognosis of HNC patients.^[8]^ Therefore, it is imperative and meaningful to find specific biomarkers and immune targets for the treatment of cancer patients.

O^6^-methylguanine-DNA methyltransferase (MGMT) is a strong prognostic biomarker in patients with glioblastoma.^[9]^ In mammals, the MGMT protein and the Mismatch Repair system play crucial roles in maintaining the stability of DNA. MGMT protein is a direct DNA repair enzyme encoded by the MGMT gene located on chromosome 10q26. It could remove O^6^-methylguanine from nucleotide chains, protecting DNA from damage caused by normal metabolic activities and toxic environmental factors. Through a stoichiometric and suicide reaction mechanism, MGMT proteins transfer the methyl group at the O6-position of guanine to the cysteine of its active site, resulting in direct restoration of the normal base and self-inactivation.^[10]^ Mismatch Repair is responsible for correcting mismatched base pairs in double-stranded DNA and restoring the normal nucleotide sequence. Mismatch Repair can be divided into 4 steps: mismatch recognition, recruitment of additional mismatch repair factors, excision of error-matched strands, and resynthesis of excised segments.^[11]^ In normal circumstances, Mismatch Repair corrects mismatched nucleotides on the newly synthesized sub-chain to ensure DNA replication fidelity. However, if the guanine on DNA chains is abnormally methylated, the O^6^-methylguanine on the DNA allows the base to bind incorrectly to cytosine or thymine, bypass the Mismatch Repair system, and continue to recombine with the mismatched base during repair, which causes gene mutation and even cell death.^[12]^ In such a mechanism, the MGMT protein could prevent DNA from O^6^-methylguanine induced mutation and provide resistance to mutagenesis.

As mentioned above, DNA methylation is an epigenetic change characterized by the modification or removal of methyl groups, which can interfere with the expression of normal genes. Hypermethylation of CpG islands in gene promoters is related to the direct silencing of tumor suppressor genes, the indirect silencing of additional gene classes and genomic instability, affecting the occurrence and development of tumors.^[13]^ As MGMT is a tumor suppressor gene, loss of MGMT expression is associated with the aggressive behavior and progression of various tumors, such as esophageal cancer, gastric cancer, hepatocellular carcinoma and lung cancer.^[14]^ However, previous studies on whether MGMT hypermethylation affects the prognosis of patients with HNC are still controversial. Some studies have reported that the expression of MGMT was decreased and the prognosis was worse in MGMT hypermethylated HNC patients.^[14,15]^ But other studies also found that there is no obvious correlation between MGMT hypermethylation and their survival rate.^[16,17]^ The inconsistent results may be due to variance in the sample size, cutoff value and experimental design of the studies.^[18]^ Hence, we conducted a meta-analysis to assess the association between MGMT hypermethylation, expression and the prognosis of HNC patients.

## 2. Methods

### 2.1. Search strategy

This meta-analysis was conducted in accordance with the Preferred Reporting Items for Systematic Reviews and Meta-Analyses 2020 guidelines and was registered at the International Prospective Register of Systematic Reviews (CRD42021274728).^[19]^ Searches were executed in PubMed, Embase, The Cochrane Library and Web of Science. All databases were searched from inception to February 1, 2023. The key words of retrieval strategy included: (“O(6)-Methylguanine-DNA Methyltransferase” OR MGMT) AND (“oral cancer” OR “tonsil cancer” OR “oropharyngeal cancer” OR “nasopharyngeal cancer” OR “laryngeal cancer” OR “hypopharyngeal cancer” OR “paranasal sinuses cancer” OR “head and neck cancer”) (see Document S1, Supplemental Digital Content, http://links.lww.com/MD/I763, which presents the full search strategies and syntaxes). Moreover, manual searches of the reference lists of retrieved articles were used to identify additional articles.

### 2.2. Inclusion and exclusion criteria

Inclusion criteria were as follows: research conducted only in humans; all patients were diagnosed with HNC; the study reported an association between MGMT and survival of HNC; provide sufficient research data including hazard ratios (HR) with prognostic endpoints and the 95% confidence interval (CI) directly, or provided other data such as Kaplan–Meier survival curves that could be used for estimating the OS; and English language publications only.

Exclusion criteria were as follows: animal experiment; the data in the studies are insufficient for meta-analysis; reviews, abstracts, case reports, letters, etc.; the data reported in the article are irrelevant to the prognosis of patients with MGMT and HNC. In the case of multiple publications covering the same cohort, the latest article was used preferentially.

### 2.3. Data extraction

The data were independently extracted and tabulated by 2 researchers (H.Y. and F.Y.) to minimize variations. If there were inconsistencies, disagreements were resolved by consulting a third researcher (J.C.). The eligible studies for this meta-analysis were independently reviewed by 2 reviewers (L.Z. and Y.W.). Data about the author, year, country, cancer type, sample size, clinical stage, gender, age, follow-up, method, survival analysis, and HR value were extracted from the studies. In this study, ethical approval was not necessary because the included data was based on previous published articles, and no original clinical data was collected or utilized. We used the Newcastle–Ottawa Quality Assessment Scale to evaluate the methodological quality of each publication, and a Newcastle–Ottawa Quality Assessment Scale ≥ 6 was considered high quality.^[20]^ The Recommendations for Prognostic Research Reports on Tumor Markers was used to assess the quality of the published reports on prognostic tumor markers.^[21]^

### 2.4. Statistical analysis

Pooled HR and 95% CI were obtained directly or estimated from the literature through *P* values and other published data according to Parmer’s method.^[22]^ The χ^2^-based *Q* test and the *I*^2^ test were used to assess heterogeneity.^[23]^ The random-effects model was used for analysis, and the fixed-effects model was used when there was no significant statistical heterogeneity between the studies (*P* > .10, *I*^2^ < 50%). We applied sensitivity analysis to investigate the impact of each individual study on the overall aggregated results. Begg’s funnel plots and Egger’s test were used to assess the publication bias. The certainty of evidence was assessed with the Grading of Recommendations Assessment, Development and Evaluation system.^[24]^ All of the statistical tests used in this meta-analysis were conducted with Stata 12.0 software (StataCorp LP, College Station, TX).

## 3. Results

### 3.1. Characteristics of the eligible studies selection process and included trial characteristics

**Figure 1.**
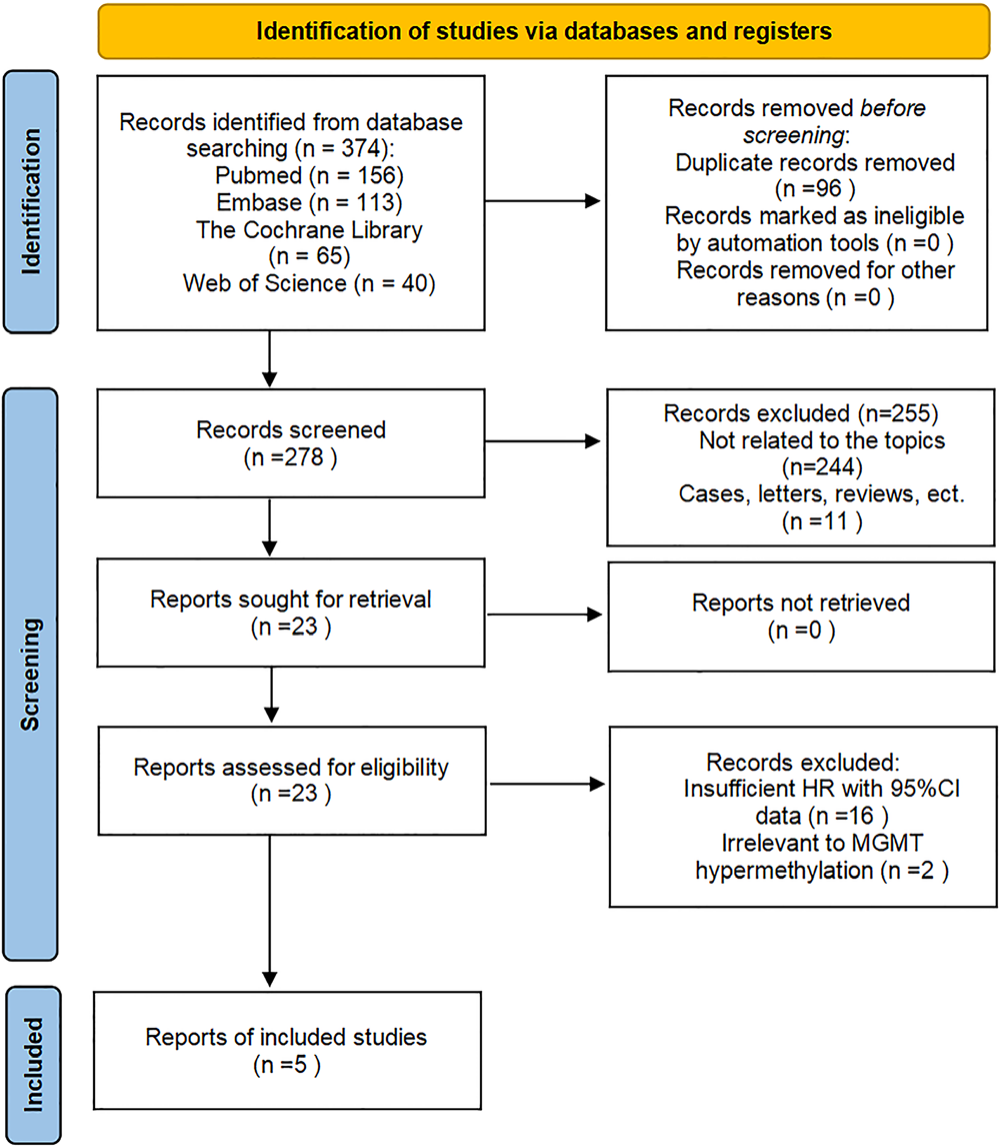
Flow chart of the study inclusion process.

A total of 374 records were retrieved by searching the electronic databases (Fig. 1). After removing duplicates, we conducted a preliminary screening of 278 articles, and based on the title and abstract of the article, 255 publications were excluded. The remaining 23 papers were screened in full text, and 18 papers were excluded: 16 were not reported sufficient HR and 95% CI data with specific prognostic endpoints, 2 was irrelevant to MGMT hypermethylation and expression. Finally, 5 articles studying 564 HNC patients were included in this meta-analysis, including two of oral squamous cell carcinomas, one of HNSCC, one of salivary gland carcinomas and one of oral or pharyngeal cancer, with publication years ranging from 2004 to 2014. The minimum sample size was 47, and the maximum was 286, as shown in Table 1. In the 5 studies that reported OS, 4 studies directly reported HR and 95% CI, and one was calculated from the Kaplan–Meier survival curve provided by the publication. Three articles reported HR and 95% CI for disease-free survival (DFS) directly.

**Table 1:** Characteristics of included studies.

Author	Year	Country	Cancer type (N)	Clinical stage (n)		Gender (male/female)	Age	Follow-up (mo)	Method	HR (95% CI)		NOS/REM-ARK score
										OS	DFS	
Zuo 1^[25]^	2004	USA	HNSCC (94)	I II III IV	26 16 16 36	94/0	63.5 (42–85)	53.4	MSP	1.66 (1.11–5.18)	2.38 (1.14–7.26)	6/15
Zuo 2^[25]^	2004	USA	HNSCC (93)	I II III IV	26 16 15 36	93/0	63.5 (42–85)	53.4	IHC	1.13 (0.74–1.13)	1.89 (0.80–6.47)	6/15
Taioli^[26]^	2009	USA	Oral or pharyngeal cancer (88)	I II III IV	20 16 17 35	56/32	62.2	65.8	MSP	2.17 (1.11–4.23)	3.49 (1.62–7.52)	6/14
Supic^[27]^	2011	Serbia	OSCC (47)	II III	16 31	33/14	58 (39–80)	26–100	MSP	1.226 (0.414–3.871)	NR	6/15
Theocharis^[14]^	2011	France	OSCC (49)	NR		26/23	60 (33–94)	40	IHC	1.14 (0.317–2.756)	1.309 (0.478–3.224)	6/15
Scesnaite^[15]^	2014	Lithuania	Salivary gland carcinomas (286)	I II III IV	66 62 62 93	136/150	NR	NR	IHC	1.238* (1.070–1.432)*	NR	7/14

DFS = disease-free survival, HNSCC = head and neck squamous cell carcinoma, HR = Hazard ratio, IHC = immunohistochemistry, MSP = methylation-specific PCR, n = number of patients, N = total number of patients, NOS = Newcastle-Ottawa Quality Scale, NR = not reported, OS = overall survival, OSCC = oral squamous cell carcinomas, REMARK = Recommendations for Tumor Marker Prognostic Studies.

* The data were calculated from the *P* value and Kaplan–Meier survival curves provided by the literature (according to the method published by Parmar et al^[22]^), but not directly obtained.

### 3.2. Association between MGMT and prognosis of HNC patients

**Figure 2.**
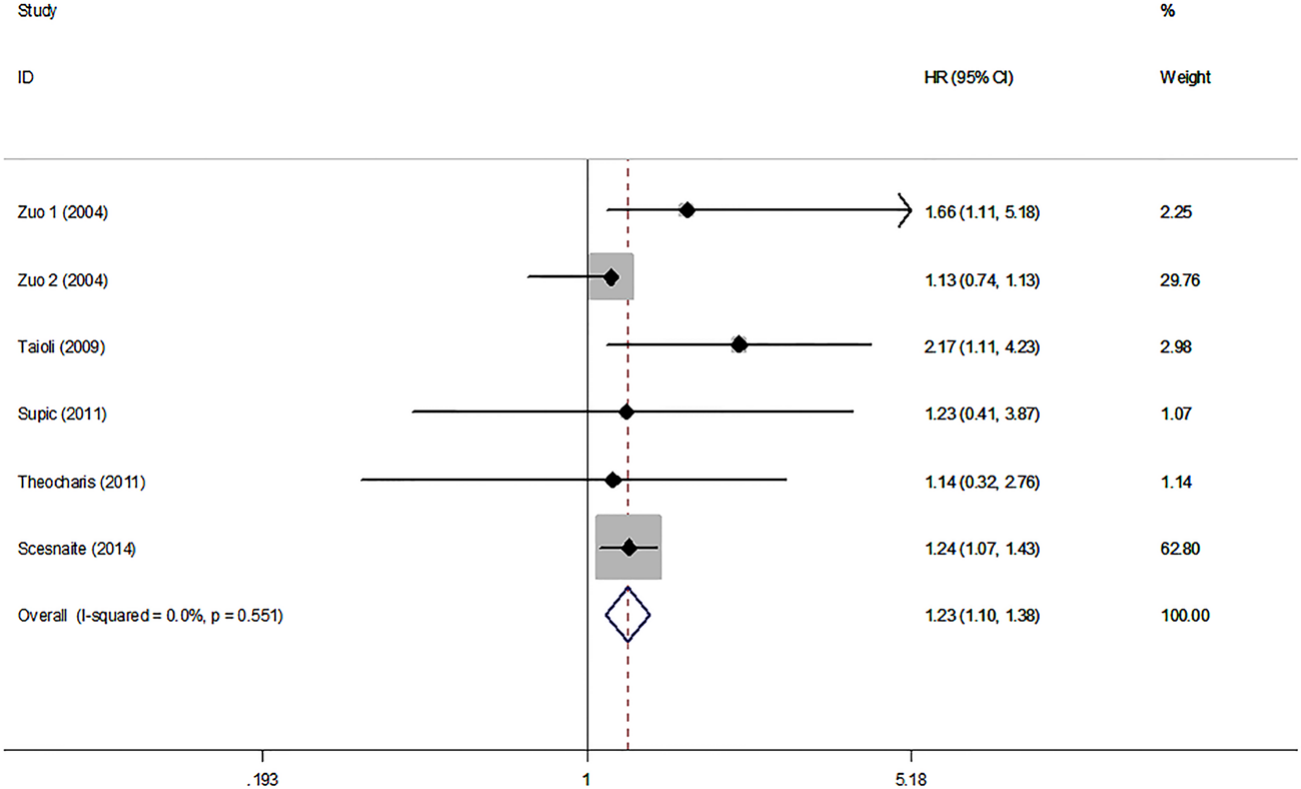
Forest plot of MGMT associated with OS in HNC patients. HNC = head and neck cancer, MGMT = O^6^-methylguanine-DNA methyltransferase, OS = overall survival.

**Figure 3.**
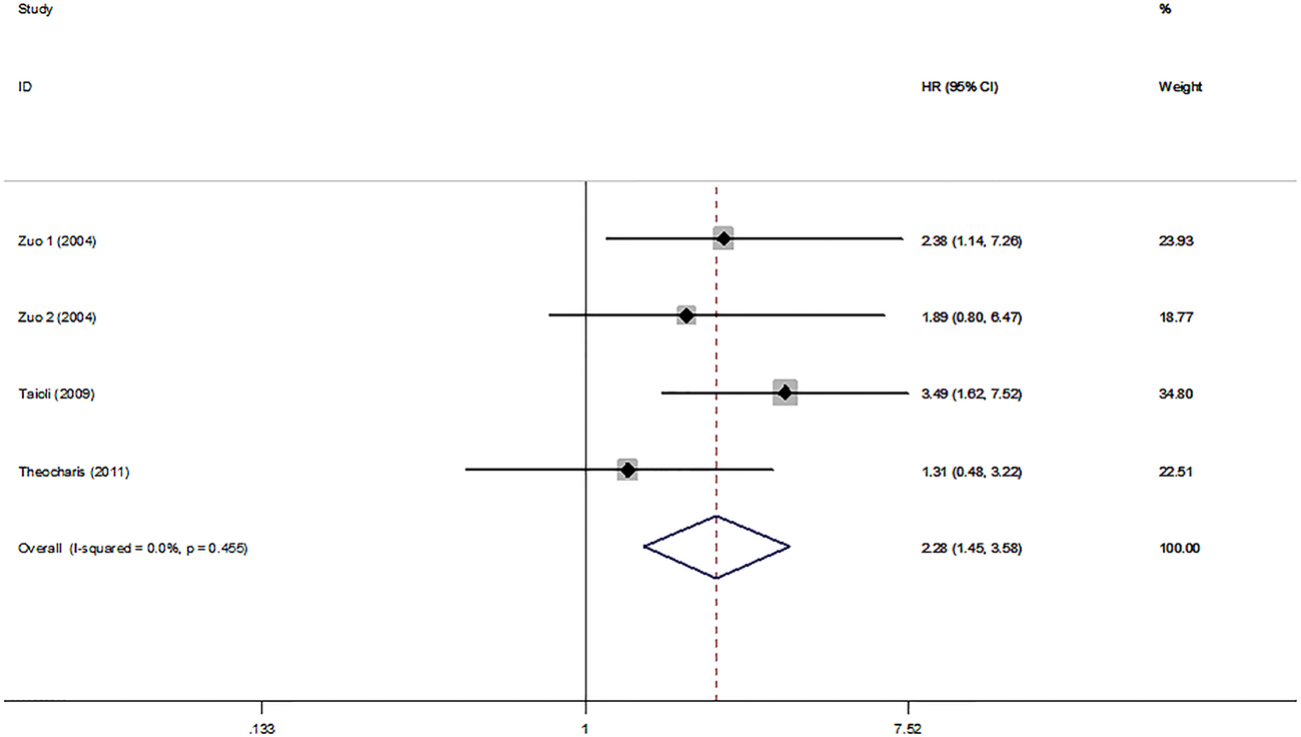
Forest plot of MGMT associated with DFS in HNC patients. DFS = disease-free survival, HNC = head and neck cancer, MGMT = O^6^-methylguanine-DNA methyltransferase.

A total of 5 studies with 564 patients were included to analyze the association between MGMT and OS in HNC patients. The results of the forest plot indicated that HNC patients with MGMT hypermethylation and low expression were associated with a poor prognosis, with a calculated HR of OS (HR = 1.23, 95% CI: 1.10–1.38, *P* < .001) (Fig. 2). Three publications with 231 HNC patients were fitted into the analysis of MGMT and DFS. In the forest plot, it can be seen that HNC patients with MGMT hypermethylation and low expression were associated with poor outcome as well, with pooled HR of DFS (HR = 2.28, 95% CI: 1.45–3.58, *P* < .001) (Fig. 3). No significant heterogeneity was noted between MGMT and OS (*I*^2^ = 0.0%, *P*_heterogeneity_ = 0.551) (Fig. 2), and no significant heterogeneity was noted between MGMT and DFS (*I*^2^ = 0.0%, *P*_heterogeneity_ = 0.455) (Fig. 3). A fixed-effects model was used. Both the certainty in the estimates of effect was judged as low due to bias and inconsistency (Table 2).

**Table 2:** Summary of finding table for the analysis of association between MGMT and prognosis of patients with HNC.

Association between MGMT and prognosis of HNC patients											
No. of studies	Certainty assessment						Effect			Certainty	Importance
	Study design	Risk of bias	Inconsistency	Indirectness	Imprecision	Other considerations	No. of events	No. of individuals	Rate (95%CI)		
Overall survival (follow-up: range 2–116 mo; assessed with: HR)											
5	Observational studies	Serious*	Serious†	Not serious	Not serious	Publication bias strongly suspected; all plausible residual confounding would suggest spurious effect, while no effect was observed*	189	564	–	⨁⨁◯◯ Low	CRITICAL
Disease-free survival (follow-up: range 2–116 mo; assessed with: HR)											
3	Observational studies	Serious*	Serious†	Not serious	Not serious	Publication bias strongly suspected; all plausible residual confounding would suggest spurious effect, while no effect was observed*	70	231	–	⨁⨁◯◯ Low	IMPORTANT

GRADE Working Group grades of evidence.

High certainty: We are very confident that the true effect lies close to that of the estimate of the effect.

Moderate certainty: We are moderately confident in the effect estimate: The true effect is likely to be close to the estimate of the effect, but there is a possibility that it is substantially different.

Low certainty: Our confidence in the effect estimate is limited: The true effect may be substantially different from the estimate of the effect.

Very low certainty: We have very little confidence in the effect estimate: The true effect is likely to be substantially different from the estimate of effect.

HNC = head and neck cancer, HR = Hazard ratio, MGMT = O6-methylguanine-DNA Methyltransferase Hypermethylation.

* The number of included articles is low, less than 10.

† In different studies, significant heterogeneity was encountered due to gender, various regimens, doses, duration, center settings, populations enrolled etc.

**Figure 4.**
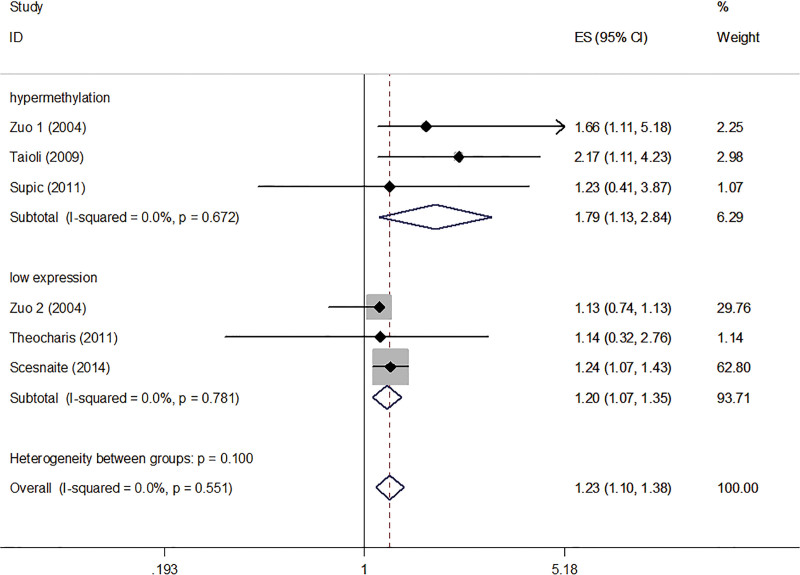
Forest plot of the OS subgroup analysis stratified by MGMT hypermethylation and low expression in HNC patients. HNC = head and neck cancer, MGMT = O^6^-methylguanine-DNA methyltransferase, OS = overall survival.

Furthermore, a subgroup analysis was conducted for HNC patients was stratified according to MGMT hypermethylation and low expression. The summarized HR for MGMT hypermethylation was 1.79 (95% CI: 1.13–2.84, *P* < .05) and for the MGMT low expression was 1.20 (95% CI: 1.07–1.35, *P* < .05). No significant heterogeneity was noted between MGMT hypermethylation and OS (*I*^2^ = 0.0%, *P*_heterogeneity_ = 0.672), and no significant heterogeneity was noted between MGMT low expression and OS (*I*^2^ = 0.0%, *P*_heterogeneity_ = 0.781) (Fig. 4).

### 3.3. Sensitivity analysis

**Figure 5.**
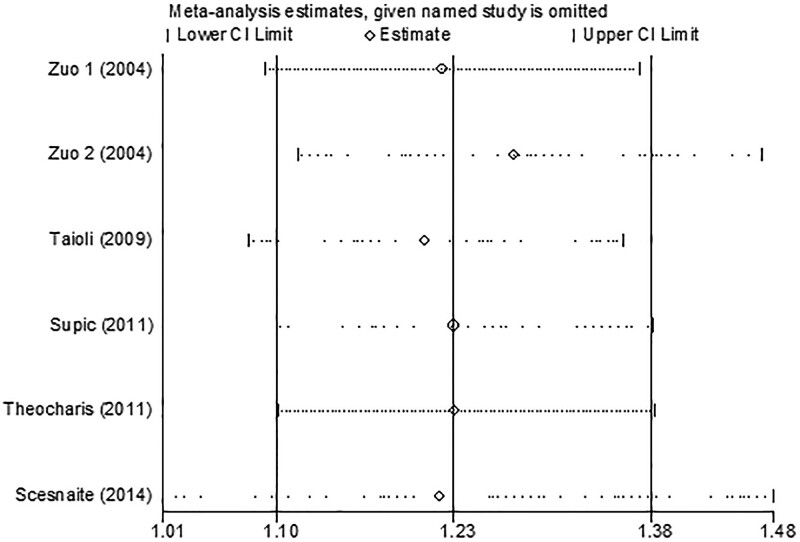
Sensitivity analyses.

Sensitivity analysis was applied to detect whether the decision-making in each step of this analysis is robust and whether it affected the results of the merger. This analysis indicated that no single study could significantly alter the combined results (Fig. 5). The results of the sensitivity analysis indicated that the pooled effect size of the meta-analysis was stable and reliable.

### 3.4. Publication bias

**Figure 6.**
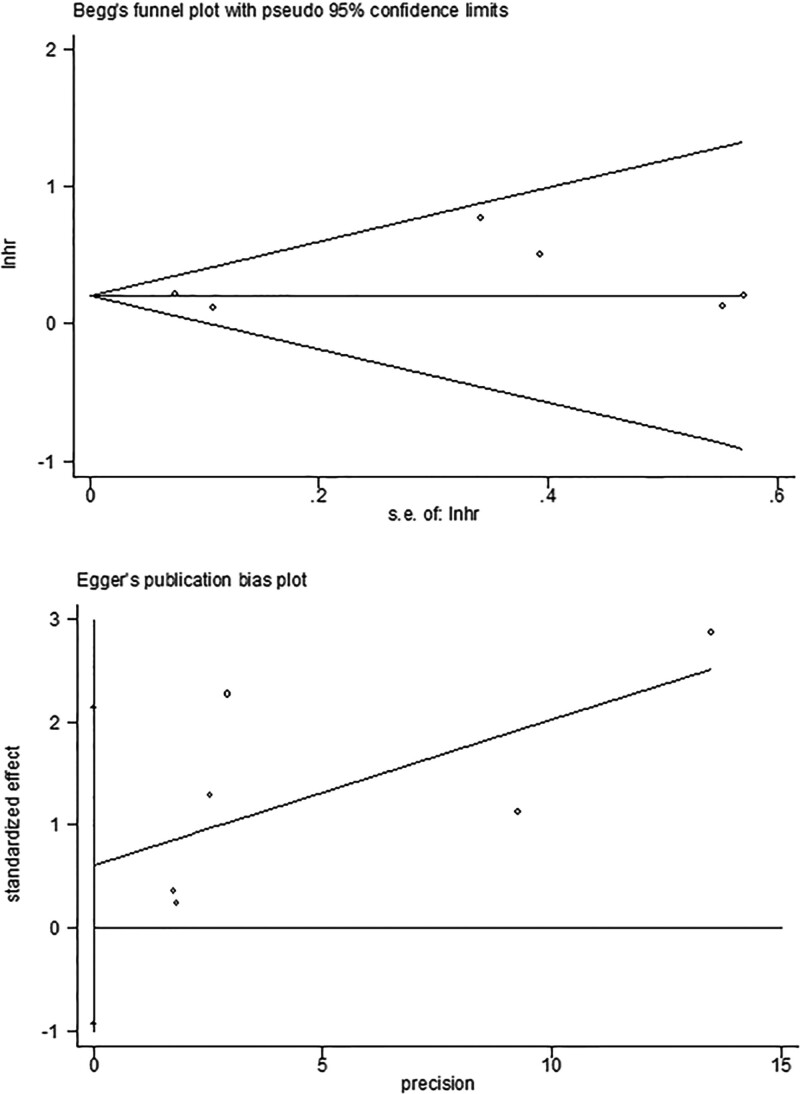
Begg’s funnel plot and Egger’s test used to assess potential publication bias of included studies.

Begg’s funnel plots and Egger’s test were applied to verify the potential bias of the included publications (Fig. 6). The results indicated that there was no publication bias (bias = 0.613; 95% CI: −0.920 to 2.147; *P* = .329). Due to the low number of included publications, the risk of publication bias cannot be excluded completely in our study.

## 4. Discussion

Aberrant methylation of the MGMT promoter is significantly associated with the risk of HNSCC^[28]^; however, researchers have different opinions about whether hypermethylated MGMT is a prognostic biomarker.^[15-17,25,29]^ Zuo et al^[25]^ studied 93 patients with HNSCC, and Scesnaite et al^[15]^ studied 286 patients with salivary gland cancer. They reported that there was a significant correlation between MGMT promoter hypermethylation and MGMT protein expression loss in HNC patients, and MGMT promoter hypermethylation and protein expression loss were significantly correlated with increased tumor recurrence and reduced patient survival, and thus they were reliable and independent prognostic factors for HNC. Furthermore, Theocharis et al^[14]^ showed that high expression of MGMT is significantly associated with a clear tumor morphology, reduced depth of invasion, and no lymph node metastasis, suggesting that MGMT may be involved in the early stages of active tongue squamous cell carcinoma, play a protective role in the body, and inhibit tumor formation and development. However, different results were found by other researchers. Dikshit et al^[17]^ found that there was no correlation between MGMT gene hypermethylation and the survival rate of laryngeal cancer and hypopharyngeal cancer patients. This is consistent with Righini’s study, which analyzed the primary tumor tissues and saliva samples of 90 patients with HNSCC and 30 matched nonmalignant head and neck lesions and did not observe any correlation between gene methylation and prognosis.^[16]^ These contradictory results may be related to the patient population and heterogeneous designs.

This study is the first meta-analysis, including 5 published studies of 564 patients, that has assessed the relationship between MGMT hypermethylation, low expression and the prognosis of HNC patients. Our results showed that HNC patients with hypermethylated and low expressed MGMT had poor OS and DFS (HR 1.23 and HR 2.28, respectively), and the results were statistically significant (Figs. 2 and 3).

**Figure 7.**
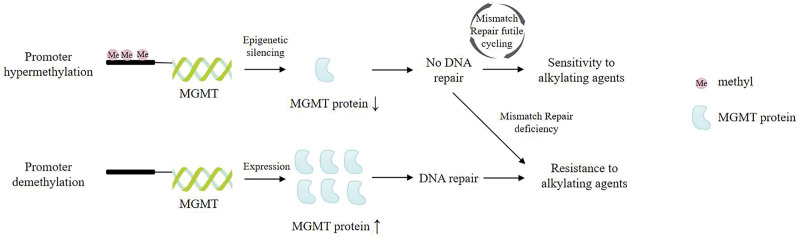
The role of MGMT promoter hypermethylation and expression in response to alkylating agents. When the MGMT promoter is hypermethylated (top), transcriptional silencing leads to low expression of MGMT protein, resulting in the lack of MGMT-mediated DNA damage repair and futile cycling of Mismatch Repair, which contributes to the DNA double-stranded break and activation of cell death. This increases sensitivity to alkylating agents in Mismatch Repair functional cells. Mismatch Repair-deficient cells do not respond to alkylating agents due to lack of Mismatch Repair-dependent DNA double-stranded breaks. When the MGMT promoter is unmethylated (bottom), cells transcribe MGMT gene and highly express MGMT protein. Alkylating adducts were removed by MGMT protein, which prompted resistance of cells to alkylating agents. MGMT = O^6^-methylguanine-DNA methyltransferase.

Alkylating agents and intercalators can form O^6^ alkyl guanine DNA adducts, deceive the mismatch repair system, affect cell division and cause cell death. Therefore, alkylating agents are used in chemotherapy for some tumors. However, MGMT can repair such DNA damage, affecting the sensitivity of tumor cells to alkylating agents such as carmustine (BCNU), temozolomide^[30]^ and streptozotocin^[31]^ (Fig. 7). In certain tumor patients with MGMT promoter methylation or with reduced MGMT gene expression, such as malignant astrocytoma, glioma and diffuse large B-cell lymphoma, alkylating chemotherapy drugs can improve their survival rate.^[25]^ This provides guidance for the use of new alkylating agents for HNC patients with MGMT promoter hypermethylation or MGMT protein deletion but with functional Mismatch Repair. There are few studies evaluating this effect currently, so more research evidence is needed to support it.

However, our current meta-analysis also has certain limitations. First, only 5 studies with 564 patients were included in the analysis, and a small sample size will increase the deviation of the final meta-analysis results. The analysis of small samples not only encountered potentially significant heterogeneity, but also suffered from significant sources of bias. Second, not all studies provided sufficient survival data. The survival data of 1 study was calculated from the presented Kaplan–Meier curve, which may not be completely accurate compared with the original data. Third, this study did not contain studies written in languages other than English, hence omitted literatures in these languages may contribute to additional bias. The vast majority of studies originated from Western countries, and there is a lack of evidence that these studies can be extrapolated to Eastern populations. Finally, the majority of the included studies reported positive results, our results might overestimate the prognostic significance of MGMT in some degree.

Although additional larger studies need to be conducted to make our results more accurate, our findings clearly lend support to this conclusion that the prognosis of HNC patients with MGMT hypermethylation and low expression is poor, MGMT has prognostic value for HNC patients and can be a potential immunotherapy target.

## Author contributions

**Data curation:** Huiwen Yang, Yanjun Wang.

Formal analysis: Yanjun Wang.

Funding acquisition: Yanjun Wang.

Software: Liuqing Zhou.

Writing – original draft: Huiwen Yang, Fan Yang.

Writing – review & editing: Jingcai Chen, Liuqing Zhou, Yanjun Wang.

## Supplementary Material
