# Supplementary material for: Prognostic value of O6-methylguanine-DNA methyltransferase hypermethylation and expression in head and neck cancer: A systematic review and meta-analysis
Source: Medicine (Baltimore). 2022 Apr 7;102(14):e33472. doi: 10.1097/MD.0000000000033472 (PMC10082309; doi:10.1097/MD.0000000000033472)
Supplement: Supplementary file 1 [file medi-102-e33472-s001.pdf]

Prognostic Value of O^6-methylguanine-DNA Methyltransferase Hypermethylation and Expression in Head and Neck Cancer: A Systematic Review and Meta-Analysis.

First author: Hui-wen Yang, Fan Yang

Search strategies and syntaxes:

Pubmed:

#1 ("O(6)-Methylguanine-DNA Methyltransferase"[Mesh]) OR ((((((((((Methylated-DNA-Protein-Cysteine S-Methyltransferase) OR (Methylated DNA Protein Cysteine S Methyltransferase)) OR (S-Methyltransferase, Methylated-DNA-Protein-Cysteine)) OR (O(6)-Methylguanine Methyltransferase)) OR (O(6)-Alkylguanine-DNA Alkyltransferase)) OR (O(6)-MeG-DNA Methyltransferase)) OR (O(6)-Methylguanine DNA Transmethylase)) OR (Guanine-O(6)-Alkyltransferase)) OR (O(6)-AGT)) OR (DNA Repair Methyltransferase II)) OR (DNA Repair Methyltransferase I))
#2 ("Head and Neck Neoplasms"[Mesh]) OR (((((((((((((((((((((((((((((((((Neoplasms, Head and Neck) OR (Head, Neck Neoplasms)) OR (Head and Neck Neoplasm)) OR (Cancer of Head and Neck)) OR (Head and Neck Cancer)) OR (Cancer of the Head and Neck)) OR (Upper Aerodigestive Tract Neoplasms)) OR (UADT Neoplasm)) OR (Neoplasm, UADT)) OR (Neoplasms, UADT)) OR (UADT Neoplasms)) OR (Neoplasms, Upper Aerodigestive Tract)) OR (Upper Aerodigestive Tract Neoplasm)) OR (Head Neoplasms)) OR (Neoplasms, Head)) OR (Head Neoplasm)) OR (Neoplasm, Head)) OR (Neck Neoplasms)) OR (Neoplasms, Neck)) OR (Neck Neoplasm)) OR (Neoplasm, Neck)) OR (Cancer of Head)) OR (Head Cancers)) OR (Head Cancer)) OR (Cancer, Head)) OR (Cancers, Head)) OR (Cancer of the Head)) OR (Cancer of Neck)) OR (Neck Cancers)) OR (Neck Cancer)) OR (Cancer, Neck)) OR (Cancers, Neck)) OR (Cancer of the Neck))
#3 ("Mouth Neoplasms"[Mesh]) OR (((((((((((((((((Mouth Neoplasm)) OR (Neoplasm, Mouth)) OR (Neoplasms, Oral)) OR (Neoplasm, Oral)) OR (Oral Neoplasm)) OR (Oral Neoplasms)) OR (Neoplasms, Mouth)) OR (Cancer of Mouth)) OR (Mouth Cancers)) OR (Oral Cancer)) OR (Cancer, Oral)) OR (Cancers, Oral)) OR (Oral Cancers)) OR (Cancer of the Mouth)) OR (Mouth Cancer)) OR (Cancer, Mouth)) OR (Cancers, Mouth))
#4 ("Tonsillar Neoplasms"[Mesh]) OR ((((((((((((((((Tonsil Neoplasms) OR (Neoplasms, Tonsil)) OR (Neoplasm, Tonsil)) OR (Tonsil Neoplasm)) OR (Neoplasms, Tonsillar)) OR (Neoplasm, Tonsillar)) OR (Tonsillar Neoplasm)) OR (Cancer of Tonsil)) OR (Tonsil Cancers)) OR (Cancer of the Tonsil)) OR (Tonsil Cancer)) OR (Cancer,

Tonsil)) OR (Cancers, Tonsil)) OR (Tonsillar Cancer)) OR (Cancer, Tonsillar)) OR (Cancers, Tonsillar)) OR (Tonsillar Cancers))
#5 ("Oropharyngeal Neoplasms"[Mesh]) OR ((((((((((((((((((Neoplasm, Oropharyngeal) OR (Oropharyngeal Neoplasm)) OR (Oropharynx Neoplasms)) OR (Neoplasm, Oropharynx)) OR (Neoplasms, Oropharynx)) OR (Oropharynx Neoplasm)) OR (Neoplasms, Oropharyngeal)) OR (Cancer of Oropharnyx)) OR (Oropharnyx Cancer)) OR (Oropharnyx Cancers)) OR (Oropharyngeal Cancer)) OR (Cancer, Oropharyngeal)) OR (Cancers, Oropharyngeal)) OR (Oropharyngeal Cancers)) OR (Oropharynx Cancer)) OR (Cancer, Oropharynx)) OR (Cancers, Oropharynx)) OR (Oropharynx Cancers)) OR (Cancer of the Oropharynx))
#6 ("Nasopharyngeal Neoplasms"[Mesh]) OR ((((((((((((((((Nasopharyngeal Neoplasm) OR (Neoplasm, Nasopharyngeal)) OR (Neoplasms, Nasopharyngeal)) OR (Nasopharynx Neoplasms)) OR (Nasopharynx Neoplasm)) OR (Neoplasm, Nasopharynx)) OR (Neoplasms, Nasopharynx)) OR (Cancer of Nasopharynx)) OR (Nasopharynx Cancers)) AND (Nasopharyngeal Cancer)) OR (Cancer, Nasopharyngeal)) OR (Cancers, Nasopharyngeal)) OR (Nasopharyngeal Cancers)) OR (Nasopharynx Cancer)) OR (Cancer, Nasopharynx)) OR (Cancers, Nasopharynx)) OR (Cancer of the Nasopharynx))
#7 ("Laryngeal Neoplasms"[Mesh]) OR ((((((((((((((((Neoplasms, Laryngeal) OR (Laryngeal Neoplasm)) OR (Neoplasm, Laryngeal)) OR (Larynx Neoplasms)) OR (Larynx Neoplasm)) OR (Neoplasm, Larynx)) OR (Neoplasms, Larynx)) OR (Cancer of Larynx)) OR (Larynx Cancers)) OR (Laryngeal Cancer)) OR (Cancer, Laryngeal)) OR (Cancers, Laryngeal)) OR (Laryngeal Cancers)) OR (Larynx Cancer)) OR (Cancer, Larynx)) OR (Cancers, Larynx)) OR (Cancer of the Larynx))
#8 ("Hypopharyngeal Neoplasms"[Mesh]) OR ((((((Hypopharyngeal Neoplasm) OR (Neoplasm, Hypopharyngeal)) OR (Neoplasms, Hypopharyngeal)) OR (Hypopharyngeal Cancer)) OR (Cancer, Hypopharyngeal)) OR (Cancers, Hypopharyngeal)) OR (Hypopharyngeal Cancers))
#9 ("Paranasal Sinus Neoplasms"[Mesh]) OR ((((((((Neoplasm, Paranasal Sinus) OR (Paranasal Sinus Neoplasm)) OR (Neoplasms, Paranasal Sinus)) OR (Paranasal Sinus Cancer)) OR (Cancer, Paranasal Sinus)) OR (Cancers, Paranasal Sinus)) OR (Paranasal Sinus Cancers)) OR (Cancer of Paranasal Sinus))
#10 #2 OR #3 OR #4 OR #5 OR #6 OR #7 OR #8 OR #9
#11 #1 AND #10

Embase:
#1 "head and neck cancer"/ or "head and neck tumor"/ or malignant neoplasms subdivided by anatomical site/ or "head and neck carcinoma"/ or larynx cancer/ or mouth cancer/ or neck cancer/ or nose cancer/ or pharynx cancer/ or tongue cancer/ or tonsil cancer/
#2 methylated dna protein cysteine methyltransferase/ or methyltransferase/
#3 #1 AND #2

The Cochrane Library:

1# Head-and-Neck-Neoplasms*:ME
2# Mouth-Neoplasms*:ME
3# Tonsillar-Neoplasm*:ME
4# Nasopharyngeal-Neoplasms*:ME
5# Oropharyngeal-Neoplasms*:ME
6# Laryngeal-Neoplasms*:ME
7# Hypopharyngeal-Neoplasms*:ME
8# Paranasal-Sinus-Neoplasms*:ME
9# ((head-and-neck) OR (0ral) OR (tonsil*) OR (oropharyn*) OR (nasopharyn*) OR (hypopharyn*) OR (laryn*) OR (paranasal sinus))
10# ((NEOPLAS*) OR (CANCER) OR (CARCINOMA*))
11# (#9 AND #10)
12# (#11 OR #1 OR #2 OR #3 OR #4 OR #5 OR #6 OR #7 OR #8)
13# O(6)-Methylguanine-DNA-Methyltransferase*:ME
14# ((O6-Methylguanine-DNA-Methyltransferase) OR (O(6)-AGT) OR (MGMT))
15# (#13 OR #14)
16# (#15 AND #12)

Web of Science:
#1 TS=("O(6)-Methylguanine-DNA Methyltransferase" OR "Methylated-DNA-Protein-Cysteine S-Methyltransferase" OR "Methylated DNA Protein Cysteine S Methyltransferase" OR "S-Methyltransferase, Methylated-DNA-Protein-Cysteine" OR "O(6)-Methylguanine Methyltransferase" OR "O(6)-Alkylguanine-DNA Alkyltransferase" OR "O(6)-MeG-DNA Methyltransferase" OR "O(6)-Methylguanine DNA Transmethylase" OR "Guanine-O(6)-Alkyltransferase" OR "O(6)-AGT" OR "DNA Repair Methyltransferase II" OR "DNA Repair Methyltransferase I")
#2 TS=("Head and Neck Neoplasms" OR "Neoplasms, Head and Neck" OR "Head, Neck Neoplasms" OR "Head and Neck Neoplasm" OR "Cancer of Head and Neck" OR "Head and Neck Cancer" OR "Cancer of the Head and Neck" OR "Upper Aerodigestive Tract Neoplasms" OR "UADT Neoplasm" OR "Neoplasm, UADT" OR "Neoplasms, UADT" OR "UADT Neoplasms" OR "Neoplasms, Upper Aerodigestive Tract" OR "Upper Aerodigestive Tract Neoplasm" OR "Head Neoplasms" OR "Neoplasms, Head" OR "Head Neoplasm" OR "Neoplasm, Head" OR "Neck Neoplasms" OR "Neoplasms, Neck" OR "Neck Neoplasm" OR "Neoplasm, Neck" OR "Cancer of Head" OR "Head Cancers" OR "Head Cancer" OR "Cancer, Head" OR "Cancers, Head" OR "Cancer of the Head" OR "Cancer of Neck" OR "Neck Cancers" OR "Neck Cancer" OR "Cancer, Neck" OR "Cancers, Neck" OR "Cancer of the Neck")
#3 TS=("Mouth Neoplasms" OR "Mouth Neoplasm" OR "Neoplasm, Mouth" OR "Neoplasms, Oral" OR "Neoplasm, Oral" OR "Oral Neoplasm" OR "Oral Neoplasms" OR "Neoplasms, Mouth" OR "Cancer of Mouth" OR "Mouth Cancers" OR "Oral Cancer" OR "Cancer, Oral" OR "Cancers, Oral" OR "Oral Cancers" OR "Cancer of the Mouth" OR "Mouth Cancer" OR "Cancer, Mouth" OR "Cancers, Mouth")
#4 TS=("Tonsillar Neoplasms" OR "Tonsil Neoplasms" OR "Neoplasms, Tonsil" OR "Neoplasm, Tonsil" OR "Tonsil Neoplasm" OR "Neoplasms, Tonsillar" OR "Neoplasm,

Tonsillar" OR "Tonsillar Neoplasm" OR "Cancer of Tonsil" OR "Tonsil Cancers" OR "Cancer of the Tonsil" OR "Tonsil Cancer" OR "Cancer, Tonsil" OR "Cancers, Tonsil" OR "Tonsillar Cancer" OR "Cancer, Tonsillar" OR "Cancers, Tonsillar" OR "Tonsillar Cancers")
#5 TS=("Oropharyngeal Neoplasms" OR "Neoplasm, Oropharyngeal" OR "Oropharyngeal Neoplasm" OR "Oropharynx Neoplasms" OR "Neoplasm, Oropharynx" OR "Neoplasms, Oropharynx" OR "Oropharynx Neoplasm" OR "Neoplasms, Oropharyngeal" OR "Cancer of Oropharnyx" OR "Oropharnyx Cancer" OR "Oropharnyx Cancers" OR "Oropharyngeal Cancer" OR "Cancer, Oropharyngeal" OR "Cancers, Oropharyngeal" OR "Oropharyngeal Cancers" OR "Oropharynx Cancer" OR "Cancer, Oropharynx" OR "Cancers, Oropharynx" OR "Oropharynx Cancers" OR "Cancer of the Oropharynx")
#6 TS=("Nasopharyngeal Neoplasms" OR "Nasopharyngeal Neoplasm" OR "Neoplasm, Nasopharyngeal" OR "Neoplasms, Nasopharyngeal" OR "Nasopharynx Neoplasms" OR "Nasopharynx Neoplasm" OR "Neoplasm, Nasopharynx" OR "Neoplasms, Nasopharynx" OR "Cancer of Nasopharynx" OR "Nasopharynx Cancers" AND "Nasopharyngeal Cancer" OR "Cancer, Nasopharyngeal" OR "Cancers, Nasopharyngeal" OR "Nasopharyngeal Cancers" OR "Nasopharynx Cancer" OR "Cancer, Nasopharynx" OR "Cancers, Nasopharynx" OR "Cancer of the Nasopharynx")
#7 TS=("Laryngeal Neoplasms" OR "Neoplasms, Laryngeal" OR "Laryngeal Neoplasm" OR "Neoplasm, Laryngeal" OR "Larynx Neoplasms" OR "Larynx Neoplasm" OR "Neoplasm, Larynx" OR "Neoplasms, Larynx" OR "Cancer of Larynx" OR "Larynx Cancers" OR "Laryngeal Cancer" OR "Cancer, Laryngeal" OR "Cancers, Laryngeal" OR "Laryngeal Cancers" OR "Larynx Cancer" OR "Cancer, Larynx" OR "Cancers, Larynx" OR "Cancer of the Larynx")
#8 TS=("Hypopharyngeal Neoplasms" OR "Hypopharyngeal Neoplasm" OR "Neoplasm, Hypopharyngeal" OR "Neoplasms, Hypopharyngeal" OR "Hypopharyngeal Cancer" OR "Cancer, Hypopharyngeal" OR "Cancers, Hypopharyngeal" OR "Hypopharyngeal Cancers")
#9 TS=("Paranasal Sinus Neoplasms" OR "Neoplasm, Paranasal Sinus" OR "Paranasal Sinus Neoplasm" OR "Neoplasms, Paranasal Sinus" OR "Paranasal Sinus Cancer" OR "Cancer, Paranasal Sinus" OR "Cancers, Paranasal Sinus" OR "Paranasal Sinus Cancers" OR "Cancer of Paranasal Sinus")
#10 (#2 OR #3 OR #4 OR #5 OR #6 OR #7 OR #8 OR #9)
#11 (#10 AND #1)
